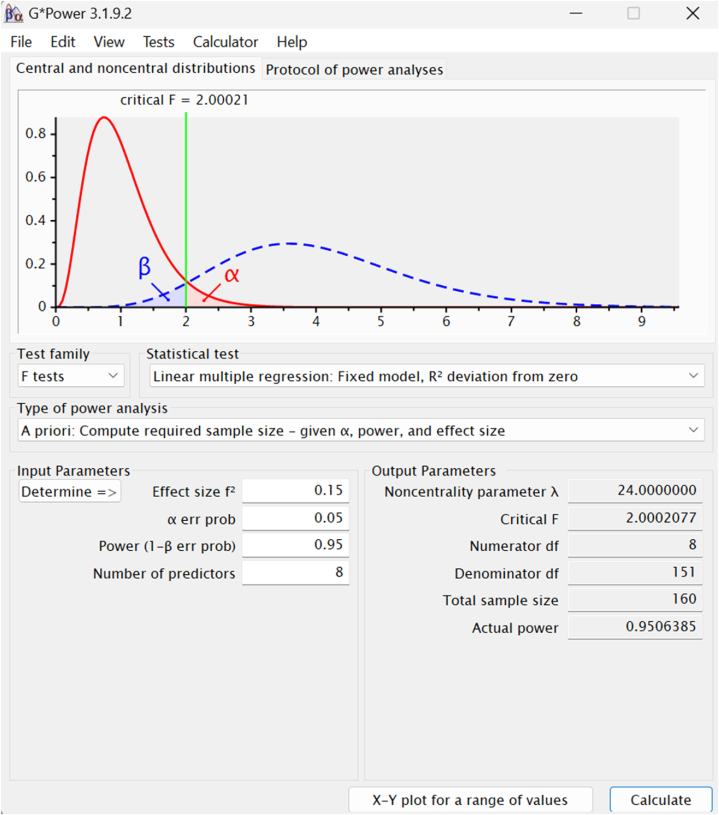# Breaking into the black box of customer perception towards robot service: Empirical evidence from service sector

**DOI:** 10.1016/j.heliyon.2024.e38117

**Published:** 2024-09-21

**Authors:** Samar Rahi, Mazuri Abd Ghani, Manaf Al-Okaily, Aamir Rashid, Mahmoud Alghizzawi, Fadi Shehab Shiyyab

**Affiliations:** a Universiti Sultan Zainal Abidin, Terengganu, Malaysia; b Hailey College of Banking & Finance, University of the Punjab, Lahore, Pakistan; c School of Business, Jadara University, Irbid, Jordan; d School of Business, The University of Jordan, Amman, Jordan; e York College, City University of New York, New York, USA; f Faculty of Business, Applied Science Private University, Amman, Jordan; g School of Business, The Hashemite University, Zarqa, Jordan

**Keywords:** Service robot, Expectation confirmation, Self-identity, Customer innovativeness, Subjective norms, Perceived behavioral control, Intention to use service robot

## Abstract

The advent of artificial intelligence and machine learning has enabled robots to serve in consumer market for a better customer experience. Nevertheless, acceptance of robotic technology among consumers is still lacking. Therefore, this study has developed an integrated model with robot appearance, expectation confirmation model, diffusion of innovation and theory of planned behavior and empirically investigates customer intention to use service robot. The research model is empirically tested with 349 responses retrieved from customers visiting retail stores. Statistical results have revealed that customer innovativeness, compatibility, behavioral control, expectation confirmation, service robot appearance and subjective norms explained $R^{2}$ 80.1 % variance in customer attitude to use service robot. Practically, this research has suggested that policy makers should pay attention in innovativeness, compatibility, perceived behavioral control, expectation confirmation, robot appearance and subjective norms to boost robot service acceptance among customers. This study is original as it develops an integrated model with the combination robot appearance, theory of planned behavior, expectation confirmation and diffusion of innovation theory. In addition to that customer self-identity is conceptualized as moderating factor and hence distinguishing current research with past studies.

## 1 Introduction

The proliferation of technology and the advent of artificial intelligence have enabled robots to perform efficiently in all kinds of industries from services to manufacturing. Moreover, the surge in technology advancement and the recent pandemic wave have changed customer perception towards the use of robots in service industry [1] Now, service robots are being used to enrich customer experiences and to achieve competitive advantage [2]. The term service robot is referred to smart autonomous and adaptable system that interacts with humans, benefits to human, communicate with them and offer enriched services to customers [3,4]. Robot service is cost-effective and can be used in banks, hotels, and airports to manage services [5].

Recently, a report issued by international federation of robotics has revealed global market value of service robot is growing to $55.72 billion by 2026 [6]. Authors like Borghi, Mariani [2] asserted that service robots have capability to analyze complex scenarios in a firm and offering unforgettable novel experiences to customers. Although robot technology has proven benefits however acceptance of robotic technology among consumers is still lacking [5]. Prior to this authors like Amelia, Mathies [7] have investigated customer acceptance of robot service in banking with thematic analysis. Nevertheless, limited research work is existed that empirically investigates customer's behavior toward acceptance robot service.

Although past studies have shed light on robot technology usage and customer acceptance of robotic services Borghi, Mariani [2], Belanche, Casaló [5], Amelia, Mathies [7], Huang and Rust [8], Grazzini, Viglia [9] however, none of them have integrated expectation conformation theory, theory of planned behavior and diffusion of innovation theory altogether to investigate consumer behavior towards acceptance and usage of robotic service. According to Rahi, Abd. Ghani [10] have argued that consumer behavior cannot be assessed with a single theory driven research model. Therefore, integration expectation conformation theory, theory of planned behavior and diffusion of innovation theory will unveil broader view of consumer behavior towards acceptance and usage of robotic service.

Aside of theory integration an important factor namely robot appearance is also studied. According to Tung and Au [11] have stated that robot appearance is as essential as staff appearance in managing quality relationship and therefore must be consider while offering robotic services. As technology is complex phenomenon and hence it is expected that customer self-identity towards robotic technology could play vital role to predict customer attitude and behavioral intention to accept service robot. Thus, moderating effect of customer self-identity is conceptualized between the relationship of customer attitude and behavioral intention to use robot service. The remaining of this manuscript follows literature, methodology, data analysis, results, discussion, research implications and conclusion.

## 2 Literature review

### 2.1 Service robot appearance and expectation confirmation

The recent innovation in service industry is the advent of robots in delivering services and enriching customer experience. Due to technology proliferation service robots have gained large attention of policy makers and being used domestically and in industry [5,12]. Robot enabled services are commonly found in warehouses to manage supply chain operations and in hotel sector to deal with security, concierge, housekeeping and delivering food to guest [8]. Prior studies have shown successful stories of service robot in managing customers and enriching visitor experience [8,9]. Although robot service provides an unforgettable experience to customers however customers have shown concern towards robot appearance [11,13]. According to Tung and Au [11] have stated that robot appearance is as essential as staff appearance in managing quality relationship. So far literature has disclosed humanoid and non-humanoid types of robots to manage services [7,13]. Authors like Amelia, Mathies [7] have stated that human like characteristics of robot like emotions and acting have more favorable impact human-robot interaction and influence attitude positively. Similarly, customer positive expectation towards robot influence customer attitude positively. Prior studies have established that consumer with mindset of robot technology would have more positive attitude towards use of robot services [[14], [15], [16], [17]]. Therefore, following hypotheses are assumed:

H1 Service robot appearance is positively related to customer attitude to use service robot.

H2 Expectation confirmation is positively related to customer attitude to use service robot.

### 2.2 Diffusion of innovation theory

In information system literature the diffusion of innovation theory has been widely used in measuring technology user's adoption behavior [[18], [19], [20], [21]]. There are mainly two core factors of DOI theory to determine user behavior towards adoption of technology namely compatibility and innovativeness [22]. Compatibility is explained the extent wherein robot technology fits to user need and work style [21]. Therefore, innovativeness denotes to any idea that is perceived by user unique and new [21]. Nevertheless, innovativeness is studies as consumer innovativeness in this study and defined as user desire to try new things for the sake of differentiation and excitement. Therefore, it is expected that service robot compatibility and user innovativeness influence user behavior to accept robot enabled services. Authors like Simões, Soares [20] have established that innovative robot technology has improved operational performance in manufacturing firms. Similarly, studies have claimed that innovative consumers are less risk conscious resulting high rate of technology adoption [14,23]. Literature has established that both user innovativeness and compatibility are identified as influential factors in adopting new technology and positively influence user attitude [14,19,23]. Following above research studies and supported by prior research Jung, Quan [14], Borghi and Mariani [19], Rahi, Alghizzawi [21], Triwijayati and Wijayanti [23] compatibility and user innovativeness are hypothesizes as:

H3 Compatibility is positively related to customer attitude to use service robot.

H4 Customer innovativeness is positively related to customer attitude to use service robot.

### 2.3 Theory of planned behavior

The theory of planned behavior has capability to examine user behavior towards adoption of new technology [22]. This theory was introduced by Ajzen [24] and has been widely studied in measuring user volitional behavior such as behavioral intention to adopt new technology. Although TPB theory has been widely extended and integrated in the context of technology acceptance Huang [25], Al-Mamary, Siddiqui [26], Albayati, Alistarbadi [27] however, little research is found that discuss how TPB factors impact customer behavior to adopt robot service [28]. There are two core factors of TPB theory namely subjective norms and behavioral control have shown positive incline towards consumer attitude to adopt new technology. The term subjective norm is identified as social pressure and others opinion on individual to perform behavior [24]. Therefore, behavioral control indicates to user perceived control to perform behavior. Prior studies have confirmed that norms rely on social information and taken as social pressure to perform a behavior that is important in others opinion [28,29]. In robotic technology context authors like Choe, Kim [28] have confirmed positive influence of restaurant customers towards acceptance of robotic restaurants. According to Hwang, Joo [29] subjective norm and behavioral control influence traveler intention to use service robot at airports. In financial sector subjective norm has shown positive influence towards user intention to adopt robot-advisor [30]. Following above arguments this study has conceptualized that subjective norm and behavioral control are positively related to customer attitude to adopt robot technology. Thus, following hypotheses are assumed:

H5 Subjective norms are positively related to customer attitude to use service robot.

H6 Perceived behavioral control is positively related to customer attitude to use service robot.

### 2.4 Customer self-identity as moderating factor

**Fig. 1:**
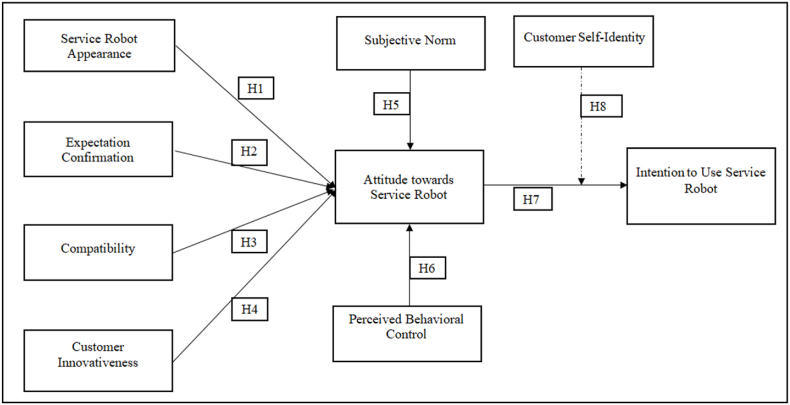
Research model.

The roots of customer self-identity can be found in self-consistency theory stated that individual tends to use services that are consistent with their self-identity [31]. Self-identity is the extent wherein customer takes action in favor of a behavior that they deem valuable for them and meet their expectations [14]. Author like Mirbabaie et al. [32], have stated that self-identity is the key motivator of individual behavior and encourage individual to perform an action. Another study conducted by Liu, Ding [31] asserted that customer intention to adopt new technology increases when self-identity match with that service. Literature has established that individual with high self-identity would have positive influence customer behavior to accept new technology [14,32,33]. Nevertheless, self-identity is conceptualized as moderating factor between customer attitude and intention to use robot service. Customer attitude is defined as degree wherein individual evaluate behavior either favorable or unfavorable towards service. Customer self-identity provides behavioral guide to customers on whether to accept or reject new technology service [31,34]. Therefore, it is assumed that customer self-identity positively moderates the relationship between customer attitude and intention to use robot services as depicted in Fig. 1. Thus, customer self-identity is hypothesized as following:

H7 Customer attitude is positively related to customer intention to use service robot.

H8 Self-identity moderates the relationship between customer attitude and customer intention to use service robot.

## 3 Research methods

### 3.1 Research design and methods

The research design of this study is grounded in positivist research paradigm. The selection of positivist research paradigm is also linked to research gap. For instance authors like Amelia, Mathies [7] have investigated customer acceptance of robot service in banking with thematic analysis. Nevertheless, limited research work is existed that empirically investigates customer's behavior toward acceptance robot service especially by following positivist research paradigm. Therefore, current research develops an amalgamated research formwork with the combination of two known theories namely theory of planned behavior and diffusion of innovation theory. As this study is conducted in services sector therefore customers visiting retail stores were considered as main population of this study. Next to this sample size is computed with priori power analysis [21,35]. Results indicate that if medium effect size 0.15 is taken with 95 % power in prior power analysis then a sample of 160 respondents is required for inferential analysis.

As the use of robot technology in services sector is new and therefore it is expected that individual would have less familiarity with robotic services. Thus, respondents were briefed first through video wherein robots are delivering services and then respondents were requested to fill survey questionnaires. This method is consistent with Choe, Kim [28] wherein they have investigated customer behavior towards acceptance of robot in restaurants. Data were collected through purposive sampling approach as recommended by prior researchers [[36], [37], [38]]. Prior to final survey pre-test was conducted with 20 respondents. In response minor contextual changes were made in scale items. For instance use of “technology” is changed to use of “service robot” for better understanding of the respondents. The main survey questionnaire was distributed among 398 customers residing in Lahore city of Pakistan. These respondents were approached physically and requested to participate in service robot survey.

Upon researcher request, 353 customers have shown interest and participated in research survey. Participation in service robot survey was completely in voluntary setting. Moreover, this study is cross sectional and therefore collects data at one point in time. Among these 353 responses 4 questionnaires were discarded due to inappropriate filling. Finally, 349 responses were retrieved from main research survey and analyzed through structural equation modeling. Prior to inferential analysis descriptive analysis is conducted. Results of the descriptive analysis have demonstrated that among 349 customers 215 were male and 134 were female. With regard to respondents age 130 respondents were identified between 41 and 50 years of age group followed by 100 respondents from 21 to 30 years of age group. Respondents who were 31–40 years old were found slightly higher (62) than 51–60 years old respondents 57. Education of the respondents was measured. Results revealed that majority of the respondents (148) were having 14 years of education followed by high school education (102) equivalent to 10 years of education. Nevertheless, 99 respondents were having 16 years of education.

### 3.2 Instrument development

Scale items were adopted from prior literature and then slightly adapted into current study context [35]. There are two main theories underpinned in research model namely theory of planned behavior and diffusion of innovation. Therefore, retrieving instrument items from literature was not a difficult. Diffusion of innovation theory comprises two core factors namely compatibility and innovativeness. Instrument items for compatibility were adapted from Ref. [18]. Therefore, customer innovativeness items were adapted from Rogers [22]. Instruments for subjective norm were adapted from Choe, Kim [28]. Perceived behavioral control instruments were adapted from Belanche, Casaló [5] and Choe, Kim [28]. Similarly, instruments for self-identity were adapted from Jung, Quan [14]. Instrument items for scale attitude towards service robot were adapted from Jung, Quan [14], Rahi and Abd Ghani [39] and Belanche, Casaló [5]. Instrument items for scale intention to use service robot were adapted from Jung, Quan [14], Rahi and Abd Ghani [5,39]. Therefore, instrument items for service robot appearance were adapted from Halkias and Diamantopoulos [9,40]. Instrument items for expectation confirmation were adapted from prior research studies [39,41]. Finally, these scale items are enumerated on 7 7-point Likert scale following a pattern that 1 indicates to disagree strongly and 7 represents to strongly agree. Table 1 exhibits scale items with respective indicator reliability, convergent validity and construct validity.

**Table 1:** Construct validity and reliability.

Scale items	Loadings	(α)	CR	AVE
Attitude towards Service Robot (ASR)				
ASR1: Offering service through robot is good idea.	0.852	0.855	0.912	0.775
ASR2: I feel pride in trying robot service.	0.892			
ASR3: I like the idea of using robot service.	0.896			
Customer Innovativeness (CIN)				
CIN1: I am willing to make experiment with robot service.	0.976	0.969	0.980	0.942
CIN2: If I hear about robot service I would like to gain its experience.	0.960			
CIN3: I would be the first to gain robot service experience among my peers.	0.975			
Compatibility (COM)				
COM1: I believe use of robot service fits well the way I like.	0.860	0.867	0.919	0.791
COM2: Use of robot service is compatible with my life style.	0.900			
COM3: Use of robot service is compatible in current situation.	0.907			
Perceived Behavioral Control (CON)				
CON1: I believe if I want I can use robot services.	0.833	0.852	0.911	0.773
CON2: I have complete control on whether to use or not use robot service.	0.895			
CON3: I am confident that if I get opportunity I will use robot service.	0.907			
Customer Self-Identity (CSI)				
CSI1: I am aware about robot service technology.	0.806	0.796	0.881	0.711
CSI2: I believe robot service is better for me.	0.838			
CSI3: I have complete knowledge about robot service.	0.885			
Expectation Confirmation (ECO)				
ECO1: Robot service feature are better than what I expected.	0.839	0.891	0.933	0.823
ECO2: My expectations towards robot service are confirmed.	0.937			
ECO3: I have positive experience with robot service technology.	0.942			
Intention to Use Services Robot (ISR)				
ISR1: I have intention to use robot service.	0.941	0.898	0.936	0.831
ISR2: I intend to use robot service in daily life.	0.880			
ISR3: Use of robot service is something that I would like to try.	0.912			
Service Robot Appearance (SAP)				
SAP1: The appearance of Service robot is friendly.	0.913	0.888	0.930	0.816
SAP2: The appearance of service robot is likable.	0.868			
SAP3: The appearance of service robot is kind and nice.	0.928			
Subjective Norm (SNO)				
SNO1: Individuals whose opinion value to me prefer to use robot service.	0.866	0.880	0.926	0.808
SNO2: other people in my circle think that I should use service robot.	0.929			
SNO3: People who are important to me think I should use robot service.	0.900			

## 4 Data analysis

### 4.1 Common method issue

The survey-based research that uses a single method for data collection could be affected by common method bias [42]. Nevertheless, this issue is addressed with Harman's single-factor solution. According to Harman's factor analysis variance explained by single factor must not be higher than 40 % [38,42]. Data were analyzed using SPSS software to get value of first un-rotated factor. Results have revealed that value of the first single un-rotated factor was 21 % i.e. less than threshold values 40 %. These findings have established that common method is not likely issue in this study and data is valid for further statistical analysis. Aside of statistical remedies researcher has followed procedural remedies to mitigate data biasness. For instance questionnaire items were jumbled before data collection. After that survey questionnaire was distributed among respondents [38]. Finally, responses retrieved from respondents were analyzed with structural equation modeling approach.

### 4.2 Structural equation modeling

The research framework has multiple variables and therefore it is tested through structural equation modeling. Hence, consistent with previous studies [[43], [44], [45], [46], [47], [48], [49], [50]] a staged approach of SEM is taken for data analysis. The first stage of SEM establishes convergent validity, indicator reliability and discriminant validity of the factors. Therefore, hypotheses are tested in second stage. Results indicate that factor loadings are higher than 0.60 demonstrating that indicators are reliable. Similarly, values of chronbach's alpha and composite reliability are higher than 0.70 indicating adequate reliability of the factors. Next to this AVE values are found higher than 0.50 demonstrating satisfactory convergent validity. The results of the first stage of structural equation modeling can be seen in Table 1.

Table 1 demonstrates satisfactory factors reliability, instrument reliability and convergent validity. Therefore, discriminant validity is measured using Fornell and Larcker analysis. According to Fornell and Larcker [51] method average variance square root value must be greater than corresponding factor correlation. Nevertheless, SEM analysis has revealed that square root value of AVE is higher than other factors and hence establishing discriminant validity of the factors. Table 2 depicts results of the Fornell and Larcker analysis.

**Table 2:** Discriminant validity.

Factors	ASR	CIN	COM	CON	CSI	ECO	ISR	SAP	SNO
ASR	0.880								
CIN	0.795	0.971							
COM	0.655	0.490	0.889						
CON	0.700	0.539	0.579	0.879					
CSI	−0.008	−0.033	0.065	0.025	0.843				
ECO	0.442	0.328	0.376	0.346	−0.085	0.907			
ISR	0.408	0.282	0.385	0.405	0.139	0.220	0.911		
SAP	0.497	0.321	0.512	0.348	0.065	0.292	0.313	0.903	
SNO	0.460	0.326	0.323	0.408	0.018	0.176	0.210	0.286	0.899

Discriminant validity of the factors is further validated with cross loading analysis as recommended by prior researchers. The cross loading analysis is actually discloses factors discriminant validity through corresponding factors loadings. The rule of thumb is that loadings of the factors must be higher than other factors loading depicting factors are discriminant. Table 3 displays loadings values of the factors and revealed that none of the factor loading is higher when comparing with other factor loadings. These findings have established discriminant validity of the factors.

**Table 3:** Cross loadings.

Factors	ASR	CIN	COM	CON	CSI	ECO	ISR	SAP	SNO
ASR1	0.852	0.934	0.528	0.571	−0.028	0.360	0.268	0.345	0.335
ASR2	0.892	0.558	0.583	0.625	0.022	0.433	0.438	0.494	0.414
ASR3	0.896	0.587	0.620	0.654	−0.014	0.375	0.376	0.480	0.470
CIN1	0.774	0.976	0.503	0.519	−0.033	0.335	0.284	0.325	0.307
CIN2	0.776	0.960	0.462	0.549	−0.038	0.319	0.270	0.320	0.321
CIN3	0.763	0.975	0.460	0.500	−0.024	0.301	0.266	0.289	0.320
COM1	0.576	0.621	0.860	0.501	0.037	0.319	0.274	0.352	0.225
COM2	0.582	0.333	0.900	0.518	0.081	0.370	0.388	0.517	0.288
COM3	0.588	0.354	0.907	0.526	0.055	0.314	0.364	0.494	0.346
CON1	0.585	0.666	0.470	0.833	0.014	0.282	0.255	0.218	0.299
CON2	0.620	0.360	0.505	0.895	0.044	0.338	0.433	0.356	0.368
CON3	0.639	0.409	0.551	0.907	0.009	0.293	0.375	0.339	0.404
CSI1	−0.026	−0.038	0.043	−0.008	0.806	−0.081	0.115	0.032	0.012
CSI2	0.032	0.014	0.081	0.055	0.838	−0.078	0.113	0.072	0.013
CSI3	−0.025	−0.056	0.041	0.018	0.885	−0.057	0.123	0.062	0.020
ECO1	0.371	0.397	0.330	0.304	−0.082	0.839	0.128	0.175	0.153
ECO2	0.425	0.247	0.350	0.312	−0.075	0.937	0.232	0.313	0.151
ECO3	0.405	0.260	0.343	0.326	−0.075	0.942	0.232	0.297	0.174
ISR1	0.346	0.259	0.335	0.363	0.124	0.141	0.941	0.249	0.190
ISR2	0.421	0.258	0.405	0.413	0.108	0.299	0.880	0.368	0.208
ISR3	0.336	0.251	0.301	0.321	0.150	0.144	0.912	0.223	0.171
SAP1	0.403	0.250	0.420	0.255	0.072	0.249	0.275	0.913	0.201
SAP2	0.508	0.364	0.514	0.390	0.053	0.250	0.278	0.868	0.325
SAP3	0.419	0.235	0.436	0.278	0.054	0.291	0.294	0.928	0.232
SNO1	0.417	0.331	0.305	0.353	0.037	0.125	0.204	0.299	0.866
SNO2	0.423	0.280	0.295	0.397	0.010	0.186	0.175	0.247	0.929
SNO3	0.400	0.266	0.270	0.347	0.001	0.162	0.187	0.225	0.900

#### 4.2.1 Hypotheses analysis

The second stage of SEM comprises the process of hypotheses testing. Hypotheses are confirmed with path coefficient values *β*, standard deviation, t-statistics and significance value. The results of the hypotheses are shown in Table 4.

**Table 4:** Hypotheses analysis.

Hypothesis	Path	*β*	STDEV	t-Statistics	Significance
H1	SAP - > ASR	0.127	0.036	3.570	0.000
H2	ECO - > ASR	0.090	0.032	2.792	0.003
H3	COM - > ASR	0.146	0.047	3.146	0.001
H4	CIN - > ASR	0.494	0.058	8.492	0.000
H5	SNO - > ASR	0.106	0.033	3.185	0.001
H6	CON - > ASR	0.230	0.057	4.048	0.000
H7	ASR - > ISR	0.411	0.060	6.870	0.000

Hypotheses analysis results have revealed that robot appearance is positively related to customer attitude to use service robot and supported by *β* = 0.127, t-statistics 3.570 significant at 0.00 and hence establishing H1. Next to this relationship between expected confirmation and customer attitude to use service robot is found significant and endorsed by *β* = 0.090, t-statistics 2.792 significant at 0.003 and hence confirmed H2. Referring to diffusion of innovation theory both factors compatibility and customer innovativeness have shown positive impact towards customer attitude to use service robot and supported by *β* = 0.146, t-statistics 3.146 significant at 0.001; *β* = 0.494, t-statistics 8.492 significant at 0.000 and hence H3 and H4 are accepted. Similarly, subjective norm and behavioral control have shown positive impact customer attitude to use service robot and reinforced by *β* = 0.106, t-statistics 3.185 significant at 0.001; *β* = 00.230, t-statistics 4.048 significant at 0.000 and hence H5 and H6 are confirmed. Finally, the relationship between customer attitude to use service robot and customer intention to use service robot is found significant and supported by *β* = 0.411, t-statistics 6.870 significant at 0.000 and hence confirmed H7.

#### 4.2.2 Factors importance performance analysis

Although research framework has established significant impact of exogenous factors in determining endogenous factors however importance of the factors is yet to be determined. Therefore, importance level is tested with importance performance matrix analysis. For IPMA analysis customer intention to use service robot is selected as outcome variable. Results have revealed that attitude has the highest importance in measuring customer intention. Therefore, customer innovativeness is the second most important factor to measure customer intention. Similarly, customer self-identity is another important factor to measure customer intention to use robot service. Thus, for policy makers this research has suggested that customer innovativeness, customer self-identity and customer attitude are most important factors which boost customer intention to use robot service. Results of the IPMA analysis are shown in Table 5 comprising importance and performance of the factors.

**Table 5:** Factors Importance and performance index.

Intention to use services robot		
Factors	Importance	Performance
Attitude towards service robot	0.411	67.508
Customer innovativeness	0.203	71.243
Compatibility	0.060	59.579
Perceived behavioral control	0.095	65.948
Customer self-identity	0.128	60.263
Expectation confirmation	0.037	61.227
Service robot appearance	0.052	60.446
Subjective norm	0.044	63.393

#### 4.2.3 Effect size and coefficient of determination

The research framework of this study is complex and therefore factors effect size $f^{2}$ is tested distinctly. There are two endogenous factors in research framework namely attitude and customer intention. Concerning with attitude results of the effect size $f^{2}$ analysis have revealed that customer innovativeness is the most important factor as it has large effect size in measuring customer attitude towards use of robot service. Therefore, perceived behavioral control is the second most important factor due to high effect size. Referring to customer intention the effect size analysis $f^{2}$ has shown large effect size of customer attitude towards intention to use service robot. Collectively, customer innovativeness, compatibility, behavioral control, expectation confirmation, service robot appearance and subjective norms explained $R^{2}$ 80.1 % variance in customer attitude i.e. substantial. Similarly, customer intention is measured with customer self-identity and customer attitude towards service robot and explained $R^{2}$ 20.1 % variance in behavioral intention to use service robot. Results of the coefficient of determination $R^{2}$ and effect size analysis are exhibited in Table 6.

**Table 6:** Factors effect sizes and coefficient of determination.

Factors	Attitude towards service robot	Effect size
Customer innovativeness	0.789	Large effect
Compatibility	0.056	Small effect
Behavioral control	0.144	Small effect
Expectation confirmation	0.033	Small effect
Service robot appearance	0.058	Small effect
Subjective norm	0.045	Small effect
Intention to use services robot		
Attitude towards service robot	0.211	Medium effect
Customer self-identity	0.020	Small effect
Coefficient of determination $R^{2}$		
Attitude towards service robot $R^{2}$	0.801	
Intention to use service robot $R^{2}$	0.201	

#### 4.2.4 Moderating analysis

**Fig. 2:**
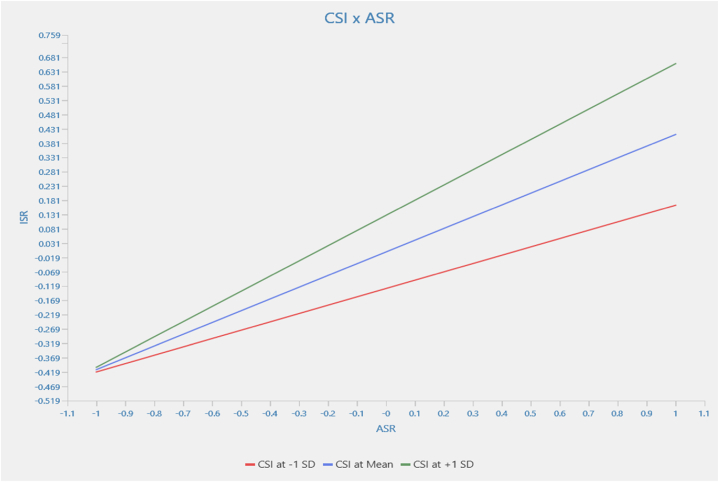
Simple slope analysis.

This study has outlined customer self-identity as moderating factor between the relationship of customer attitude and customer intention to use service robot. Data were analyzed using product indicator approach [21]. Results indicate that self-identity positively moderates the relationship between customer attitude and intention to use service robot and statistically supported by *β* = 0.120, t-statistics 1.855 significant at p 0.032 hence establishing H8. Appendix 1 exhibits values of moderating factors including t-statistics and path coefficient. Moreover, simple slope analysis as depicted in Fig. 2 shows strength of the relationship. Result shows an upwards trend of CS1 at +1SD indicating increase in customer self-identity will strengthen the relationship between customer attitude and customer intention to use service robot.

## 5 Discussion

The robot technology is found remarkable technology and delivering offering of services in commercial markets. For instance robots have successfully performed their duties as robot nurses in hospitals during pandemic and robot receptionist in hotels and departmental stores [7]. Nevertheless, use of robot service in real-world is still in its initial stages. Therefore, current study highlights factors which influence individual attitude and intention to use robot enabled services. The research model has combined factors underpinned diffusion of innovation theory, theory of planned behavior, expectation confirmation and robot appearance. Results indicate that robot appearance is positively related to customer attitude to use service robot and consistent with prior studies [11,13]. Similarly, expectation confirmation is positively related to customer attitude to use service robot and consistent with prior research work [14,15]. Moving further compatibility and customer innovativeness have shown positive impact towards customer attitude to use service robot and in line with prior studies [14,23,[52], [53], [54], [55], [56]]. These findings have established that robot technology compatibility with individual tasks and innovativeness positively influence customers attitude and encourage customers to use robot service. The research framework has examined impact of subjective norm and behavioral control in determining customer attitude. Results demonstrate that subjective norm positively influence customer attitude to use service robot and consistent with prior studies [28,29]. Similarly, perceived behavioral control has shown positive impact customer attitude and consistent with prior research work [30]. Extending to this the relationship between customer attitude to use service robot and customer intention to use service robot is found significant and in line with previous studies [31,34,57]. Moving further the moderating effect of customer self-identity between attitude and intention to use was found significant and consistent with prior arguments established by prior researchers [14,32,33,[58], [59], [60], [61]]. The simple slope analysis has revealed the strength of the relationship. Results demonstrate that with higher level of customer self-identity will strengthen the relationship between customer attitude and behavioral intention to use service robot. Aside of hypotheses acceptance research model has shown substantial variance in outcome variable. Results have revealed that collectively customer innovativeness, compatibility, behavioral control, expectation confirmation, service robot appearance and subjective norms had explained $R^{2}$ 80.1 % variance in customer attitude. These findings have established validity of the research model in measuring customer behavioral intention towards use of robot services.

## 6 Contributions

### 6.1 Research contribution to theory and methods

This research has extensively contributed to literature especially in the context of robotic technology. For instance research framework has incorporated core dimension of expectation confirmation/disconfirmation model namely expectation confirmation and established significant impact of expectation conformation towards customer attitude. Therefore, establishing positive impact of expectation conformation in measuring customer attitude contributes to literature. Similarly, this research has confirmed that innovativeness and compatibility positively impact customer attitude to use robot enabled services and enrich information system literature. Moreover, subjective norm and behavioral control have shown positive impact in determining customer attitude to use service robot and hence contributes to IS literature. Aside of theories integration another important factor namely robot appearance is tested in research model. Results have established that robot appearance impact customer attitude and increase behavioral intention to use robot service and hence contribute to robot technology literature. Another theoretical contribution of this research is to confirm moderating effect of customer self-identity between the relationship of customer attitude and intonation to use robot service. In terms of methodology this research has selected sample size through priori power analysis and hence contributes to methods. Output of priori power analysis can be seen in Appendix 2. For data computation structural equation modeling approach is employed. Thus, incorporating latest statistical analysis including SEM, effect size analysis, importance performance matrix index and priori power analysis significantly contributes to methods.

### 6.2 Research contribution to practice

The current study has several contributions to practical field. For instance results indicate that customer innovativeness, compatibility, behavioral control, expectation confirmation, service robot appearance and subjective norms are core factors to determine customer attitude in acceptance of robot services and therefore need policy maker's attention. As robot enabled service is a completely new invention in industry therefore help is taken through importance performance index to visualize importance and performance of the factors. Results of the importance performance index analysis have indicated that customer innovativeness, customer self-identity and attitude are most influential factors which boost customer intention to use robot service. Moreover, effect size $f^{2}$ analysis have revealed that policy makers should pay attention towards customer innovativeness and perceived behavioral control which in turn motivate individual to use robot enabled services. In addition to that customer self-identity is found another influential moderating factor in this study. This study has revealed that customer self-identity towards robotic technology will boost user attitude and behavioral intention to use service robot. Thus, it is essential that customers must have familiarity with robot enabled services. Therefore, if policy makers want to achieve maximum performance through limited resource they must pay attention towards achieving customer innovativeness, perceived behavioral control and customer self-identity.

## 7 Conclusion

The advent of service robot has significantly improved firm capabilities to provide lower cost services and enriching customer experience. Nevertheless, acceptance of service robot is at its initial stages due to complex nature of technology. Therefore, current study aims to investigate factors which impact customer intention to use service robot. This study has developed an integrated model with robot appearance, expectation confirmation model, diffusion of innovation and theory of planned behavior and empirically investigates customer intention to use service robot. This study is original as it develops an integrated model with the combination robot appearance, theory of planned behavior, expectation confirmation and diffusion of innovation theory. Results have revealed that customer innovativeness, compatibility, behavioral control, expectation confirmation, service robot appearance and subjective norms explained $R^{2}$ 80.1 % variance in customer attitude to use service robot. Therefore, customer intention is measured with customer self-identity and customer attitude towards service robot and explained $R^{2}$ 20.1 % variance in behavioral intention to use service robot. More precisely the effect size $f^{2}$ analysis has disclosed substantial effect size of customer innovativeness in measuring customer attitude to use robot service. Moreover, IPMA analysis has revealed that customer attitude has the highest importance in measuring customer intention to use service robot. Therefore, customer innovativeness is the second most important factor to measure customer intention. Similarly, customer self-identity is another important factor to measure customer intention to use robot service. Aside of direct relationship of customer identity this study has also established that customer self-identity moderates the relationship between customer attitude and intention to use service robot. In terms of research contribution this research contributes to literature as it integrates expectation confirmation, diffusion of innovation and theory of planned behavior in the context of robotic technology. Practically, this research has suggested that policy makers should pay attention in innovativeness, compatibility, perceived behavioral control, expectation confirmation, robot appearance and subjective norms to boost robot service acceptance among customers.

### 7.1 Limitations and future directions

In spite of several theoretical, methodological and practical contributions this study has some limitations and therefore acknowledged to impute future research directions. For instance research framework of this study has outlined single factor of expectation confirmation theory namely expectation conformation. Nevertheless, other factors of expectation confirmation theory like, disconfirmation, usefulness and satisfaction may be considered in future studies. Moreover, this study is lacking cultural aspect and therefore extending current research model with cultural factor could reveal interesting results. Another limitation of this study is that it focuses on customer acceptance and initial usage of service robot technology among customers. Nevertheless, further studies may be conducted to understand customer actual usage behavior and continuance intention to sue service robot. Similarly, the context of this research is general and respondents are from retail stores. Nevertheless, future researchers may investigate consumer behavior towards acceptance of robot service in a specific sector like use of robotic technology in banks. This study is cross sectional and examine robot service phenomenon in current scenario. Nonetheless, understanding consumer behavioral with longitudinal data set could reveal interesting results. Finally, replicating current research model in developed country could enhance generalizability of the current research framework.

## Availability of data and material

Authors confirm that all relevant data are included in this manuscript, and all sources are well cited.

## Ethical statements

All subjects gave their informed consent for inclusion before they participated in the study.

## Funding

This research received no external funding.

## CRediT authorship contribution statement

**Samar Rahi:** Writing – original draft, Data curation, Conceptualization. **Mazuri Abd Ghani:** Writing – review & editing, Validation, Resources. **Manaf Al-Okaily:** Investigation, Formal analysis. **Aamir Rashid:** Writing – review & editing, Supervision, Project administration. **Mahmoud Alghizzawi:** Writing – original draft, Funding acquisition, Formal analysis. **Fadi Shehab Shiyyab:** Writing – review & editing, Software, Methodology.

## Declaration of competing interest

The authors declare the following financial interests/personal relationships which may be considered as potential competing interests:MANAF AL-OKAILY reports a relationship with Jadara University that includes: board membership. Corresponding Author “Manaf Al- Okaily” is Associate Editor in Heliyon Journal If there are other authors, they declare that they have no known competing financial interests or personal relationships that could have appeared to influence the work reported in this paper. If there are other authors, they declare that they have no known competing financial interests or personal relationships that could have appeared to influence the work reported in this paper.

## Figures and Tables

**Figure:**
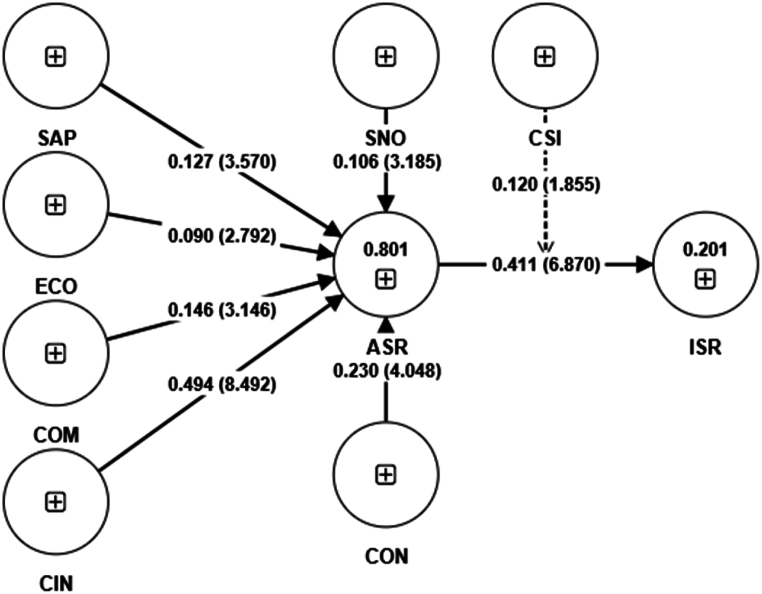


**Figure:**